# A novel method of caenophidian snake sex identification using molecular markers based on two gametologous genes

**DOI:** 10.1002/ece3.3057

**Published:** 2017-05-22

**Authors:** Nararat Laopichienpong, Panupong Tawichasri, Lawan Chanhome, Rattanin Phatcharakullawarawat, Worapong Singchat, Attachai Kantachumpoo, Narongrit Muangmai, Sunutcha Suntrarachun, Kazumi Matsubara, Surin Peyachoknagul, Kornsorn Srikulnath

**Affiliations:** ^1^ Laboratory of Animal Cytogenetics and Comparative Genomics (ACCG)Department of GeneticsFaculty of ScienceKasetsart UniversityBangkokThailand; ^2^ Animal Breeding and Genetics Consortium of Kasetsart University (ABG ‐ KU)BangkokThailand; ^3^ Snake FarmQueen Saovabha Memorial InstituteThe Thai Red Cross SocietyBangkokThailand; ^4^ Mildpets Animal HospitalBangkokThailand; ^5^ Center for Advanced Studies in Tropical Natural ResourcesNational Research University‐Kasetsart UniversityThailand (CASTNARNRU‐KUThailand)Kasetsart UniversityBangkokThailand; ^6^ Department of Fishery BiologyFaculty of FisheriesKasetsart UniversityBangkokThailand; ^7^ Department of Research and DevelopmentQueen Saovabha Memorial InstituteThe Thai Red Cross SocietyBangkokThailand; ^8^ Research Center for Bioinformatics and BiosciencesNational Research Institute of Fisheries ScienceJapan Fisheries Research and Education AgencyYokohamaKanagawaJapan; ^9^ Department of BiologyFaculty of ScienceNaresuan UniversityPhitsanulokThailand

**Keywords:** *CTNNB1*, gametolog, sex chromosome, snake, *WAC*

## Abstract

Sex identification provides important information for ecological and evolutionary studies, as well as benefiting snake conservation management. Traditional methods such as cloacal probing or cloacal popping are counterproductive for sex identification concerning very small species, resulting in difficulties in the management of their breeding programs. In this study, the nucleotide sequences of gametologous genes (*CTNNB1* and *WAC* genes) were used for the development of molecular sexing markers in caenophidian snakes. Two candidate markers were developed with the two primer sets, and successfully amplified by a single band on the agarose gel in male (ZZ) and two bands, differing in fragment sizes, in female (ZW) of 16 caenophidian snakes for *CTNNB1* and 12 caenophidian snakes for *WAC*. Another candidate marker was developed with the primer set to amplify the specific sequence for *CTNNB1*W homolog, and the PCR products were successfully obtained in a female‐specific 250‐bp DNA bands. The three candidate PCR sexing markers provide a simple sex identification method based on the amplification of gametologous genes, and they can be used to facilitate effective caenophidian snake conservation and management programs.

## 1 INTRODUCTION

Sex identification is important for mating systems and sexual behavior in ecological and conservation research in vertebrates. Intriguingly, vertebrates display considerable diversity in their sex determination systems, especially in squamate reptiles which have both temperature‐dependent sex determination (TSD) and genotypic sex determination (GSD) with ZZ/ZW‐type, XX/XY‐type, or multiple (X_1_X_2_Y and Z_1_Z_2_W) sex chromosomes (Ezaz, Srikulnath, & Graves, 2017). Moreover, sex identification cannot be reliably determined in many squamate reptiles, because males and females have similar morphology, and morphological sexual dimorphism appears in the short period of time before mating (Frýdlová et al., 2011; Garland, 1985; Wangkulangkul, Thirakhupt, & Voris, 2005). Snakes (Serpentes) are a species‐rich lineage of extant reptiles that exhibit phenotypically diverse radiation (Castoe, Jiang, Gu, Wang, & Pollock, 2008; Castoe et al., 2009; Secor & Diamond, 1998), with distribution broadly found in arboreal, terrestrial, and aquatic habitats. The snakes are considered as excellent model organisms for biomedical research, and snake venom is extracted for developing antivenoms to treat snakebites or for chemotherapeutical development (Blackburn, 2006; Kerkkamp et al., 2016; Ratanabanangkoon et al., 2016). Two distinct groups of snakes are classified as follows: (i) Scolecophidia including “blind” snakes, and (ii) Alethinophidia comprising Henophidia (pythons, boas, and other “primitive” snakes) and Caenophidia (advanced snakes) (Pyron, Burbrink, & Wiens, 2013; Wiens et al., 2012). Snake ecology, population, and diversity have been widely studied (Laopichienpong et al., 2016, 2017; Liu et al., 2015; Supikamolseni et al., 2015). Accurate sex identification is important for snake management and breeding. Improvements in conservation programs are necessary to identify the sex of juveniles before the development of primary and secondary sexual characteristics to reduce the risk of extinction, or when samples are obtained without handling individuals (e.g., noninvasive sampling). This is a very important feature when working with endangered species (Waits & Paetkau, 2005). Sex identification of snakes is commonly conducted by observation of sexually dimorphic characters such as size between sexes, with males usually having larger body, length of tail, and body color as basal practical procedure (Laszlo, 1975). However, males and females have similar morphologies in several snake species such as *Acrochordus* spp., thereby making it difficult to identify sexes in snakes (Wangkulangkul et al., 2005). This has led to the development of alternative sexing methods based on observation of the sex organ (hemipenes). Several methods have been developed to observe the sex organs, such as cloacal probing or cloacal popping (Laszlo, 1975). However, these methods induce stress with potential injury and require specific skills. There is a crucial need for the development of a rapid, safe, and accurate method for sex identification in snakes.

Molecular and chromosomal processes underlying the sex determination of vertebrates have been extensively investigated, especially in mammals and birds (Ezaz et al., 2017). The homologous gene located in the nonrecombining region of differentiated sex chromosomes is known as a “gametologous gene.” In mammals, these are the *ZFX* and *ZFY* genes and in birds they are the *CHDZ* and *CHDW*, and *ATP5A1Z* and *ATP5A1W* genes which can be applied to determine the sex of individuals (Carmichael, Fridolfsson, Halverson, & Ellegren, 2000; Ellegren & Fridolfsson, 1997; García‐Moreno & Mindell, 2000; Lawson & Hewitt, 2002). Almost all caenophidian snakes exhibit GSD with ZZ/ZW‐type sex chromosomes. Z sex chromosomes which have homology with chicken chromosomes 2 and 27 is the fourth or fifth largest metacentric chromosome in the karyotypes of most snake species (Matsubara et al., 2006, 2012; Rovatsos, Vukić, Lymberakis, & Kratochvíl, 2015; Vicoso, Emerson, Zektser, Mahajan, & Bachtrog, 2013). Recently, sequence analysis of two gametologous genes as the catenin (cadherin‐associated protein) beta 1 (*CTNNB1*) and the WW domain containing adaptor with coiled‐coil (*WAC*) has provided information on the evolutionary process of sex chromosome differentiation in snakes (Laopichienpong et al., 2017; Matsubara, Nishida, Matsuda, & Kumazawa, 2016; Matsubara et al., 2006). Size and sequence differences between the Z and W homologs of these genes were found in many caenophidian snakes, leading us to develop molecular sexing markers to identify male and female individuals. In this study, we developed novel molecular sexing markers using three primer pairs to amplify fragments from the Z and W homologs of the genes with a clear size difference of PCR products. This assay provides a rapid and reliable method to identify genetic sex across different caenophidian snake species.

## 2 MATERIALS AND METHODS

### 2.1 Specimen and DNA extraction

Twenty‐two snake species containing both male and female individuals were examined, and detailed information was presented in Table 1. The sex of each species was identified morphologically and confirmed by mating observations and sexing probes that searched for the male hemipenes. Blood samples were collected from the ventral tail vein using a 25‐gauge needle attached to a 1‐ml disposable syringe containing 10 mm ethylenediaminetetraacetic acid (EDTA). Whole genomic DNA was extracted following the standard salting‐out protocol as described previously (Supikamolseni et al., 2015) and used as templates for polymerase chain reaction (PCR). DNA quality and concentration were determined using 1% agarose gel electrophoresis and spectrophotometric analysis. Animal care and all experimental procedures were approved by the Animal Experiment Committee, Kasetsart University, Thailand (approval no. ACKU00359) and conducted according to the Regulations on Animal Experiments at Kasetsart University.

**Table 1:** Molecular sexing markers of 22 snake species with both males and females

Species	Families	Abbreviation	*CTNNB1*				Accession number	*WAC*				Accession number	*CTNNB1W* specific				No. of used snakes
			DNA band patterns	Ta	Size (bp)			DNA band patterns	Ta	Size (bp)			DNA band patterns	Ta	Size (bp)		
					Male	Female				Male	Female				Male	Female	
*Cylindrophis ruffus*	Cylindrophiidae	CRU	++	55	750	750	LC076763, LC076764	++	63	1,200	1,200	LC213910, LC213921	−	65			1 male, 1 female
*Epicrates maurus*	Boidae	EMA	++	55	750	750	LC213019, LC213020	++	63	600	600	LC213911, LC213912	−	65			1 male, 1 female
*Xenopeltis unicolor*	Xenopeltidae	XUN	++	55	750	750	LC076761, LC076762	++	63	2000	2000	LC213922, LC213923	−	65			2 males, 2 females
*Python bivittatus*	Pythonidae	PBI	++	55	750	750	LC076765, LC076766	++	63	2000	2000	LC213924, LC213925	−	65			1 male, 1 female
*Python regius*	Pythonidae	PRE	++	55	750	750	LC213021, LC213022	++	63	2000	2000	LC213926, LC213927	−	65			4 males, 2 females
*Acrochordus javanicus*	Acrochordidae	AJA	++	55	1,000	1,000	LC213023, LC213024	+++	63	2000	3,500, 1,000	LC213928, LC213929, LC213930	−	65			1 male, 1 female
*Daboia siamensis*	Viperidae	DSI	+++	55	1,100	1,100, 700	LC076767, LC076768, LC076769	+++	63	1,200	2,200, 1,200	LC213931, LC213933, LC213932	+	63		250	1 male, 1 female
*Enhydris enhydris*	Homalopsidae	EEN	+++	55	3,000	3,000, 700	LC213904, LC213905, LC213918	+++	63	1,000	1,200, 1,000	LC213934, LC213913, LC213935	+	65		250	2 males, 2 females
*Naja kaouthia*	Elapidae	NKA	+++	55	1,100	1,100, 700	LC076789, LC076790, LC076791	++	63	1,000	1,000	LC213936, LC213937	+	63		250	1 male, 1 female
*Naja siamensis*	Elapidae	NSI	+++	55	1,100	1,100, 700	LC076792, LC076793, LC076794	++	63	1,000	1,000	LC213938, LC213939	+	63		250	2 males, 1 female
*Ophiophagus hannah*	Elapidae	OHA	+++	55	1,200	1,200, 700	LC076795, LC076796, LC076797	++	63	1,000	1,000	LC213940, LC213941	+	63		250	2 males, 1 female
*Bungarus candidus*	Elapidae	BCA	+++	55	1,100	1,100, 700	LC076798, LC086064, LC086065	++	63	1,000	1,000	LC213942, LC213943	+	63		250	1 male, 1 female
*Bungarus flaviceps*	Elapidae	BFL	+++	55	1,100	1,100, 700	LC213025, LC213026, LC213027	++	63	1,000	1,000	LC213944, LC213945	+	63		250	1 male, 1 female
*Leioheterodon madagascariensis*	Lamprophiidae	LMA	+++	55	1,100	1,100, 700	LC213028, LC213029, LC213030	+++	63	1,000	1,000, 800	LC213946, LC213947, LC213948	+	65		250	1 male, 1 female
*Oligodon fasciolatus*	Colubridae	OFA	+++	55	1,500	1,500, 700	LC076772, LC076773, LC076774	+++	63	1,000	1,200, 1,000	LC213949, LC213914, LC213950	+	65		250	2 males, 1 female
*Ahaetulla prasina*	Colubridae	APR	+++	55	1,500	1,500, 700	LC076775, LC076776, LC076777	+++	63	2,500	2,500, 800	LC213951, LC213952, LC213915	+	63		250	2 males, 2 females
*Boiga dendrophila*	Colubridae	BDE	+++	55	1,700	1,700, 700	LC076778, LC076779, LC076780	+++	63	1,000	1,000, 800	LC213953, LC213954, LC213955	+	65		250	2 males, 2 females
*Gonyosoma oxycephalum*	Colubridae	GOX	+++	55	1,300	1,500, 700	LC076781, LC076782, LC076783	+++	63	2,500	2,500, 1,300	LC213916, LC213917, LC213956	+	63		250	1 male, 1 female
*Coelognathus flavolineatus*	Colubridae	CFL	+++	55	1,500	2000, 700	LC076784, LC076785, LC076786	+++	63	1,000	1,000, 800	LC213957, LC213958, LC213959	+	63		250	1 male, 1 female
*Coelognathus radiatus*	Colubridae	CRA	+++	55	2,200	2,200, 700	LC213906, LC213907, LC213919	+++	63	1,000	1,000, 800	LC213960, LC213961, LC213962	+	63		250	3 males, 5 females
*Ptyas mucosa*	Colubridae	PMU	+++	55	1,500	1,500, 700	LC213908, LC213909, LC213920	+++	63	1,000	1,500, 1,000	LC213963, LC213965, LC213964	+	63		250	2 males, 1 female
*Pantherophis guttatus*	Colubridae	PGU	+++	55	1,500	1,500, 700	LC213031, LC213032, LC213033	+++	63	1,000	1,000, 800	LC213966, LC213967, LC213968	+	63		250	6 males, 3 females

“−” No DNA band in both male and female.

“+” One DNA band in female only.

“++” One DNA band in both male and female.

“+++” One DNA band in male and two DNA bands in female.

### 2.2 Molecular sexing marker development

For *WAC* genes, partial DNA fragments of exons 9–10 were amplified using target‐specific primers Eq‐WAC‐int9‐F: 5′‐CTCAGCCATCTAATCAGTCCCCAA‐3′ and Eq‐WAC‐int9‐R: 5′‐GAACGCTGAAGACTTCGAGGAG‐3′ (Matsubara et al., 2016). For the *CTNNB1* gene, partial DNA fragments were amplified using PCR primers (Eq‐CTNNB1‐11‐F1: 5′‐AGAGACGTCCACAATCGGATTG‐3′ and Eq‐CTNNB1‐13‐R: 5′‐CAGACGTTTCTTATAATCTTGTGG‐3′) (Laopichienpong et al., 2017; Matsubara et al., 2016). The female‐specific PCR products with different size from the DNA fragments commonly found for both males and females (*WACZ* and *CTNNB1Z*) were expected to derive from the genes *WACW* and *CTNNB1W* (File S1). In addition, a new female‐specific primer, CTNNB1W‐F: 5′‐GACAAAGAAGCAGCTGAGTCAG‐3′, was designed, based on the nucleotide difference between the *CTNNB1Z* and *CTNNB1W* sequences at exon 11 identified in our previous study (Laopichienpong et al., 2017), to amplify a female‐specific PCR product using CTNNB1W‐F and Eq‐CTNNB1‐13‐R (File S1). The *BDNF* (brain derived neurotrophic factor) gene was used as a positive PCR control marker using primers BDNF‐F (5′‐GACCATCCTTTTCCTGACTATGGTTATTTCATACTT‐3′) and BDNF‐R (5′‐CTATCTTCCCCTTTTAATGGTCAGTGTACAAAC‐3′) (Leaché & McGuire, 2006). PCR amplification was performed using 20 μl of 1× ExTaq buffer containing 1.5 mm MgCl_2_, 0.2 mm dNTPs, 5.0 μm the primers, and 0.25 U of TaKaRa Ex Taq (TaKaRa Bio, Otsu, Japan), and 25 ng of genomic DNA. PCR conditions was as follows: an initial denaturation at 94°C for 3 min, followed by 35 cycles of 94°C for 30 s, 55°C for 30 s, and 72°C for 1 min, and a final extension at 72°C for 7 min. The nucleotide sequences of the DNA fragments derived from the PCR reaction of the primer sets Eq‐CTNNB1‐11‐F1 and Eq‐CTNNB1‐13‐R or Eq‐WAC‐int9‐F and Eq‐WAC‐int9‐R were only determined directly using the DNA sequencing services of First Base Laboratories Sdn Bhd (Seri Kembangan, Selangor, Malaysia). Nucleotide sequences of the Z and W homologs from each species were searched for homologies with the nucleotide sequences in the National Center for Biotechnology Information (NCBI) database to identify DNA fragments of the target gene, using the BLASTx and BLASTn programs. All the sequences were then deposited in the DNA Data Bank of Japan (DDBJ) (Table 1).

## 3 RESULTS

**Figure 1:**
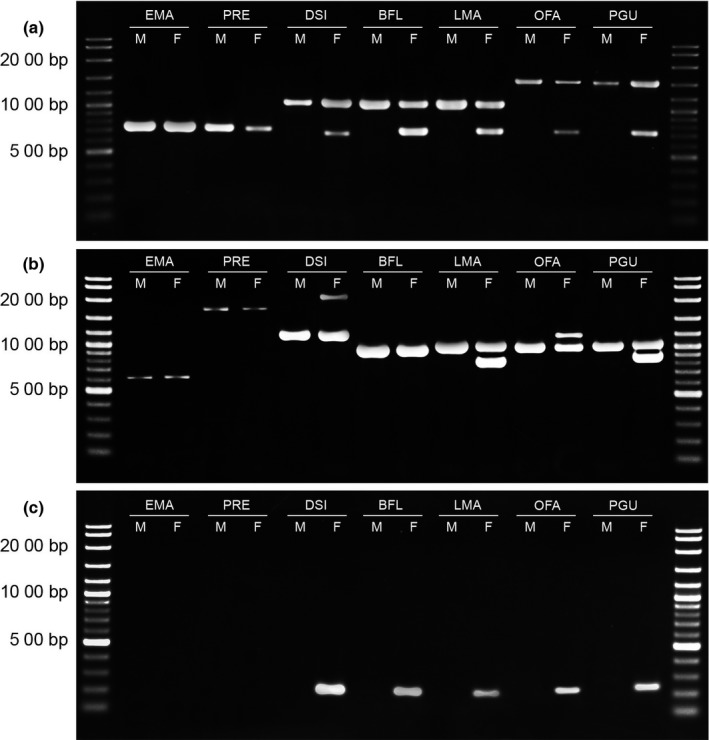
Agarose gel electrophoresis of PCR products in males and females of seven snake species using Eq‐CTNNB1‐11‐F1 and Eq‐CTNNB1‐13‐R (a), Eq‐WAC‐int9‐F and Eq‐WAC‐int9‐R (b), and CTNNB1W‐F and Eq‐CTNNB1‐13‐R (c). Molecular size of DNA is indicated in the left lane using VC 100‐bp Plus DNA ladder (Vivantis Technologies Sdn Bhd, Selangor Darul Ehsan, Malaysia). EMA,* Epicrates maurus*; PRE,* Python regius*; DSI,* Daboia siamensis*; BFL,* Bungarus flaviceps*; LMA,* Leioheterodon madagascariensis*; OFA,* Oligodon fasciolatus;* PGU,* Pantherophis guttatus*. M, male F, female

**Figure 2:**
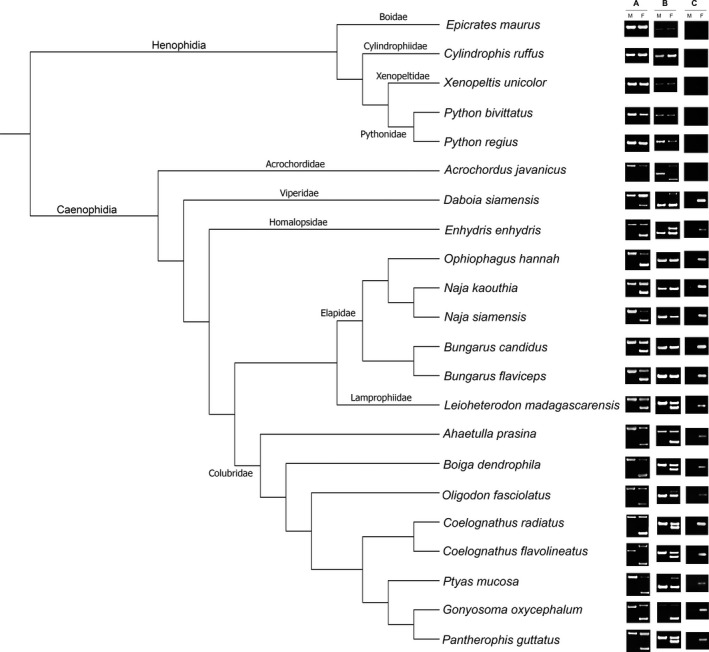
Phylogenetic relationships among sampled snake species illustrating the sex‐specific amplification of *CTNNB1* and *WAC* genes using the primers: Eq‐CTNNB1‐11‐F1 and Eq‐CTNNB1‐13‐R; Eq‐WAC‐int9‐F and Eq‐WAC‐int9‐R; and CTNNB1W‐F and Eq‐CTNNB1‐13‐R. Phylogeny was partially derived from Vidal, Rage, Couloux, and Hedges (2009). Agarose gel electrophoresis of PCR products in males and females of twenty‐two snake species using three sexing markers: Eq‐CTNNB1‐11‐F1 and Eq‐CTNNB1‐13‐R (column A), Eq‐WAC‐int9‐F and Eq‐WAC‐int9‐R (column B), and CTNNB1W‐F and Eq‐CTNNB1‐13‐R (column C) are indicated at the right edge of the tree. M, male; F, female

By agarose gel electrophoresis with the primer Eq‐CTNNB1‐11‐F1 and Eq‐CTNNB1‐13‐R, one same‐sized DNA fragment was observed in both males and females, and one additional DNA fragment was detected from females in each snake species: *Daboia siamensis*,* Enhydris enhydris*,* Naja kaouthia*,* N. siamensis*,* Ophiophagus hannah*,* Bungarus candidus*,* B. flaviceps*,* Leioheterodon madagascariensis*,* Oligodon fasciolatus*,* Ahaetulla prasina*,* Boiga dendrophila*,* Coelognathus radiatus*,* Ptyas mucosa*, and *Pantherophis guttatus*. This indicates that the same‐sized DNA bands and the female‐specific DNA bands were derived from the Z and W homologs, respectively. No female‐specific DNA fragments were observed in *Cylindrophis ruffus*,* Epicrates maurus*,* Xenopeltis unicolor*,* Python bivittatus*,* P. regius*,* Acrochordus javanicus*,* Gonyosoma oxycephalum*, and *C. flavolineatus* (Table 1; Figures 1, 2; Files S2–S4). However, there was a wide variation in the DNA fragment sizes between species, ranging from approximately 750 bp in *C. ruffus*,* E. maurus*,* X. unicolor*,* P. bivittatus*, and *P. regius* to 3,000 bp in *E. enhydris* for *CTNNB1Z* (Table 1). Individual size variation within the same species was also found in the Z‐derived fragments in two species: *G. oxycephalum* and *C. flavolineatus* (Table 1). In addition to the results from PCR with Eq‐CTNNB1‐11‐F1 and Eq‐CTNNB1‐13‐R primers, the primer pairs Eq‐WAC‐int9‐F and Eq‐WAC‐int9‐R yielded one similar‐sized DNA fragment for both males and females, and one additional DNA fragment for females in each snake species: *D. siamensis*,* E. enhydris*,* L. madagascariensis*,* O. fasciolatus*,* A. prasina*,* B. dendrophila*,* G. oxycephalum*,* C. flavolineatus*,* C. radiatus*,* P. mucosa,* and *P. guttatus*, but no female‐specific DNA fragments were observed in *C. ruffus*,* E. maurus*,* X. unicolor*,* P. bivittatus*,* P. regius*,* A. javanicus*,* N. kaouthia*,* N. siamensis*,* O. hannah*,* B. candidus,* and *B. flaviceps*. A wide size variation between species was found for both the Z‐ and W‐derived fragments. The Z‐derived fragments ranged from approximately 600 bp in *E. maurus* to 3,500 bp in *A. javanicus*, and the W‐derived fragments range from 800 bp in *L. madagascariensis*,* A. prasina*,* B. dendrophila*,* C. flavolineatus*,* C. radiatus*, and *P. guttatus* to 2,200 bp in *D. siamensis* (Table 1). Individual size variation within the same species was also found in Z‐derived fragments in one species: *A. javanicus*.

Additionally, in PCR with the *CTNNB1W*‐specific primer set, amplification of DNA fragments was found only in females as a single 250‐bp DNA band (Figures 1 and 2). To distinguish the absences of PCR products in males from failures of PCR reactions, the *BDNF* primers were used separately as an internal control under the same PCR condition. The *BDNF* primers produced larger fragments than the *CTNNB1W*‐specific products. Samples were identified as female if two products of 250‐bp (*CTNNB1W*) and 750‐bp sized bands (*BDNF*) were observed and male if only the control band was amplified (Figure 2; File S5). If the control locus failed to amplify, then sex was not assigned.

## 4 DISCUSSION

Sex identification is very important, not only for the basic understanding of the ecology and behavior of endangered or protected animals but also for establishing management and conservation plans (Dubey et al., 2011; Webb, Brook, & Shine, 2002). To contribute to breeding programs, the sexes of the snakes must be precisely identified while avoiding snake injury and stress. Traditional methods of cloacal probing or cloacal popping are counterproductive in very small species, whereas PCR has the advantage of identifying sexually heteromorphic PCR products as genomic DNA from small quantities of tissue such as blood in this study, or applied for skin remnants, slough, or eggshell membranes left behind after hatching as shown in birds (Martín‐Gálvez et al., 2011). Based on the hypothesis of sex chromosome differentiation, the cessation of recombination between sex chromosomes leads to the accumulation of gene mutations, resulting in the occurrence of sexually antagonistic alleles or the functional inactivation of genes, followed by the partial deletion of the sex chromosomes (Ezaz et al., 2017). In snake lineages, Boidae and Pythonidae have morphologically homomorphic Z and W chromosomes (Matsubara et al., 2006; Olmo & Signorino, 2005). By contrast, the W chromosomes are highly degenerated in caenophidian snakes that predominantly showed heteromorphic ZW sex chromosomes (Beçak & Beçak, 1969; Beçak, Beçak, & Nazareth, 1964; Matsubara et al., 2006, 2016; O'Meally et al., 2010; Oguiura, Collares, Furtado, Ferrarezzi, & Suzuki, 2009; Ray‐Chaudhuri, Singh, & Sharma, 1971; Singh, 1972; Vicoso et al., 2013). Considering the practically routine technique, cytogenetic approaches to examine the sex chromosomes require large sample volume with long cell culture and chromosome preparation time. Therefore, they are not practical in terms of wildlife ecological studies and conservation programs. A molecular sexing method utilizing sex‐specific sequences is, thus, more advantageous than cytogenetic analyses to identify sex chromosome systems. We developed novel PCR‐based molecular sexing methods with three primer sets to identify individual caenophidian snake sex, based on the nucleotide sequence differences of two gametologous genes. In molecular sexing with two of the three sets, Eq‐CTNNB1‐11‐F1 and Eq‐CTNNB1‐13‐R, and Eq‐WAC‐int9‐F and Eq‐WAC‐int9‐R, the males with the homogametic sex chromosome (ZZ) were characterized by a single DNA fragment band from the two Z homologs, and the females with the heterogametic sex chromosome (ZW) were identified by two bands differing in fragment sizes from the one Z and one W homologs. The two primer sets Eq‐CTNNB1‐11‐F1—Eq‐CTNNB1‐13‐R and Eq‐WAC‐int9‐F—Eq‐WAC‐int9‐R were available for molecular sexing in 16 and 12 caenophidian snakes, respectively. These two markers exhibited co‐dominant DNA pattern type. This suggests that the Z and W forms of the *CTNNB1* or *WAC* genes were differentiated by the cessation of recombination in these caenophidian lineages, which led to insertions and deletions of nucleotide sequences on the Z and W chromosomes.

No female‐specific PCR products of W homolog were found in *A. javanicus* for the *CTNNB1* gene with the primer set Eq‐CTNNB1‐11‐F1 and Eq‐CTNNB1‐13‐R and *N. kaouthia*,* N. siamensis*,* O. hannah*,* B. candidus*, and *B. flaviceps* for the *WAC* gene with the primer set Eq‐WAC‐int9‐F and Eq‐WAC‐int9‐R, even though these species belong to Caenophidia. This suggests that the absence of the size differences with female‐specific PCR products was caused by the accumulation of mutations at existing primer sites, leading to failure of PCR reaction at the W homologs. Moreover, large insertions in the amplified target fragments could result in failure of PCR amplification for the extended W sequences. Alternatively, *CTNNB1* and *WAC* may have been lost independently from the W chromosomes in the species. This result agreed with the chromosome and genomic map of snakes which showed the absence of these two genes in some snake lineages (Matsubara et al., 2006; Vicoso et al., 2013). The third explanation is that there is none or little difference in sizes of PCR products between the Z and W homologs, as a consequence of a very small region of the nonrecombining portion of the W chromosome which did not have time to diverge significantly from the Z chromosome in these genes. Snake W sex chromosomes are known to degenerate at varying rates and undergo substantial reorganization over short periods of evolutionary time (Oguiura et al., 2009). The observation of DNA bands of the two primer sets revealed high variability in length between species or individuals, which probably determined individual sex difficulty (Table 1; Figure 1). Such individual variation in the Z form of the gametologous gene within the same species was also reported by nucleotide analysis for the *CHD1Z* gene of birds (Casey, Jones, Sandercock, & Wisely, 2009; Friesen, Congdon, Walsh, & Birt, 1997; Trimbos et al., 2013) and for the *CTNNB1* gene of snakes (Laopichienpong et al., 2017).

To avoid the misidentification of sexes through size variation, we designed the additional primer at the exon of the *CTNNB1W* gene which yielded a female‐specific 250‐bp PCR products in 16 caenophidian snakes and indicated dominant DNA pattern type. Internal PCR control with the *BDNF* gene was also used to avoid amplification failure which might result in a misidentification of a female as a male. The present results of the three sexing markers could also be applied to identify the sex of individuals in new snake species as a simple procedure for males and females. We highlight that all the three sexing markers, with an additional *BDNF* control marker, could be simultaneously used to identify sex with higher diagnostic accuracy.

In the two henophidian snakes *P. bivittatus* and *B. constrictor*,* CTNNB1* and *WAC* genes were located on both Z and W chromosomes, and no sex‐specific sequence was detected for the two gametologous genes (Matsubara et al., 2006, 2016; Vicoso et al., 2013). In this study, five henophidian snakes (*C. ruffus*,* E. maurus*,* X. unicolor*,* P. bivittatus*, and *P. regius*) showed the absence of female‐specific PCR products in molecular sexing with our three primer sets. This implies that the *CTNNB1* and *WAC* genes on the Z and W chromosomes have not been differentiated in henophidian snakes (Laopichienpong et al., 2017; Matsubara et al., 2006, 2016; Vicoso et al., 2013). However, more intensive sampling over a wider range of individuals or species within the same family is required to confirm the absence of *CTNNB1* and *WAC* genes on the W chromosome. Simple PCR assay could not identify the sex of species with homomorphic sex chromosomes or highly similar Z and W sex chromosomes. Advanced molecular techniques are required to investigate cryptic sex‐specific sequences in these species.

Currently, only two PCR methods have been described to determine the sex in caenophidian snakes. Firstly, a simple PCR assay is used to amplify at the gametologous genes *CTNNB1* and *WAC* on the Z and W chromosomes that showed size difference of DNA bands, or the simple presence/absence sex‐linked marker or a specific allele based on nucleotide substitution. The alternative approach uses a single set of primers with a quantitative real‐time PCR (qPCR) technique to amplify Z‐specific genes without homologs in the W chromosome (Rovatsos & Kratochvíl, 2017; Rovatsos et al., 2015). Males (ZZ) have twice as many copies of genes linked to the Z‐specific part of sex chromosomes than females (ZW), while genes in autosomal or pseudoautosomal regions should have equal copy numbers in both sexes. Although qPCR techniques offer a high‐throughput environment for routine genotyping, the cost of the qPCR reaction is high compared to the simple PCR assay and needs extra equipment. By contrast, simple PCR assay provides a rapid, reliable, sensitive, cost‐effective, and highly accurate result for sex identification. However, both the two PCR methods cannot be used for species with poorly differentiated sex chromosomes.

This study reveals the potential and usefulness of gametologous sequences which develop new candidate sexing markers in caenophidian snakes. Our work opens a new tool kit for further application with the genomic DNA of small and degraded tissues such as slough as noninvasive sampling. To facilitate application of this tool, we provide a “know‐how” guide to apply Loop‐mediated lsothermal Amplification (LAMP) or aptamer to conservation program where species identification or sex determination is needed in real time and *in situ*. Future work in this direction will enormously facilitate the work of wildlife managers, researchers, and exotic snake breeders with important conservationist, economic, and commercial benefits.

## CONFLICT OF INTEREST

None declared.
