# Supplementary figures and images for: A novel method of caenophidian snake sex identification using molecular markers based on two gametologous genes
Source: Ecol Evol. 2017 May 22;7(13):4661–9. doi: 10.1002/ece3.3057 (PMC5496543; doi:10.1002/ece3.3057)

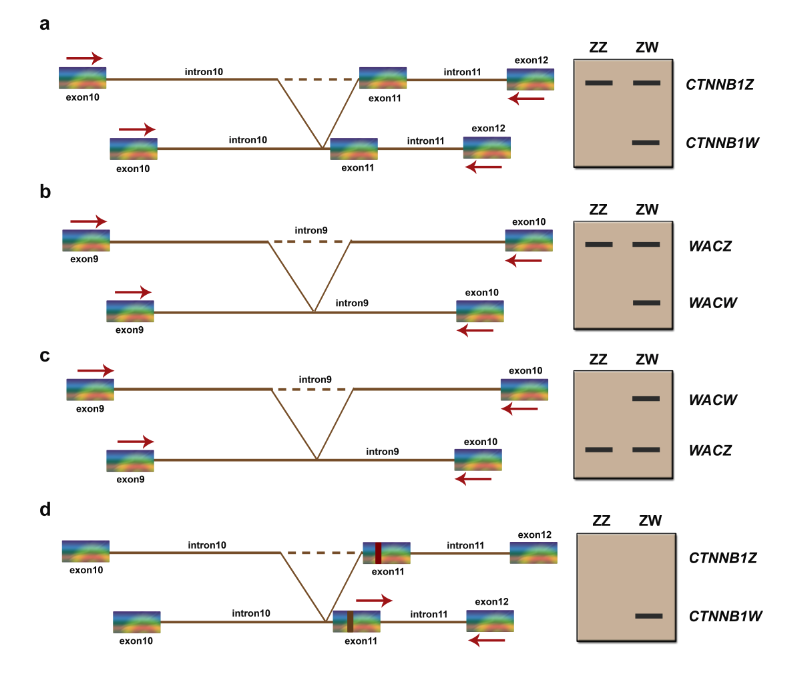

Supplement: Supplementary file 1 [file ECE3-7-4661-s001.png]

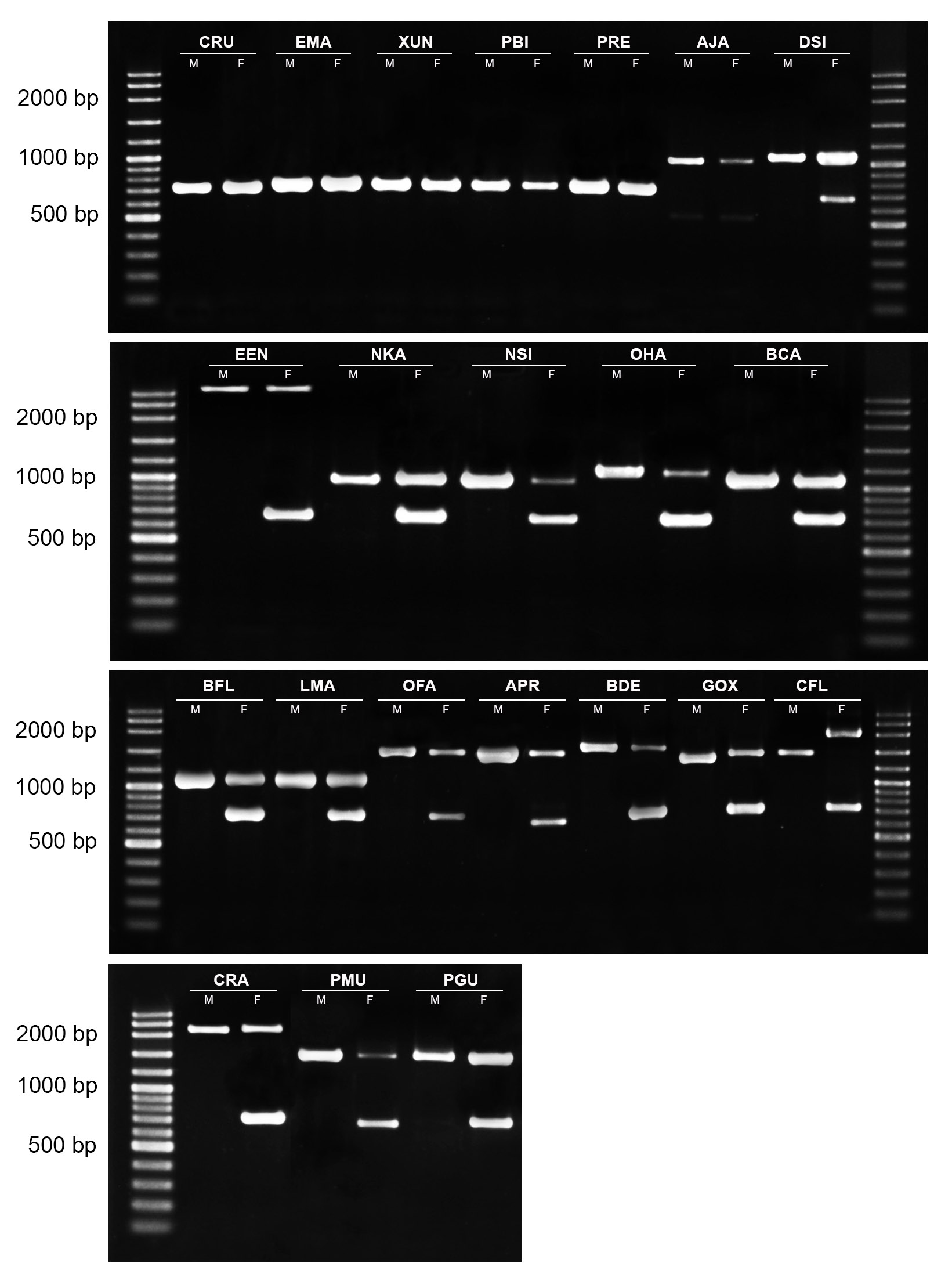

Supplement: Supplementary file 2 [file ECE3-7-4661-s002.jpg]

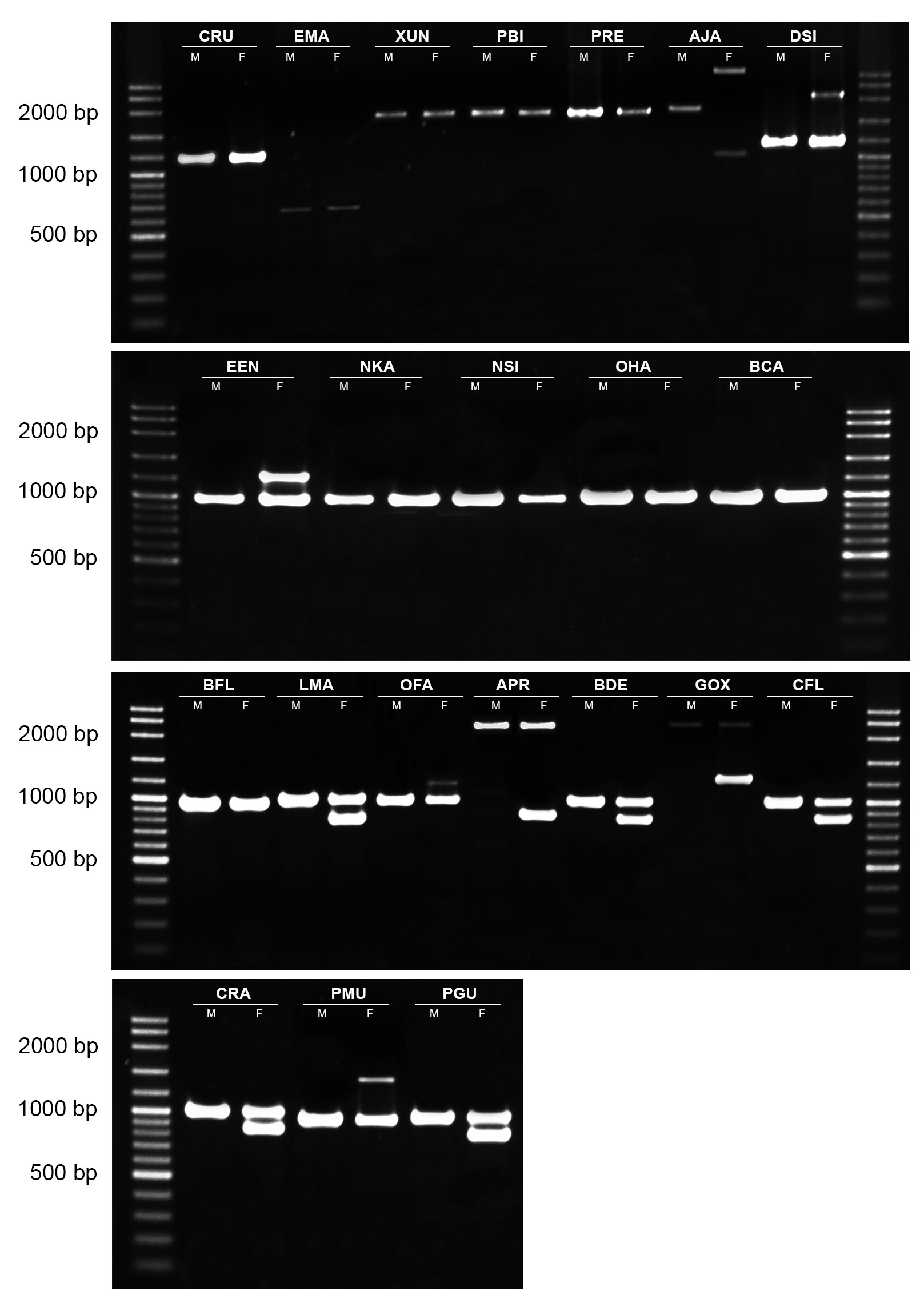

Supplement: Supplementary file 3 [file ECE3-7-4661-s003.jpg]

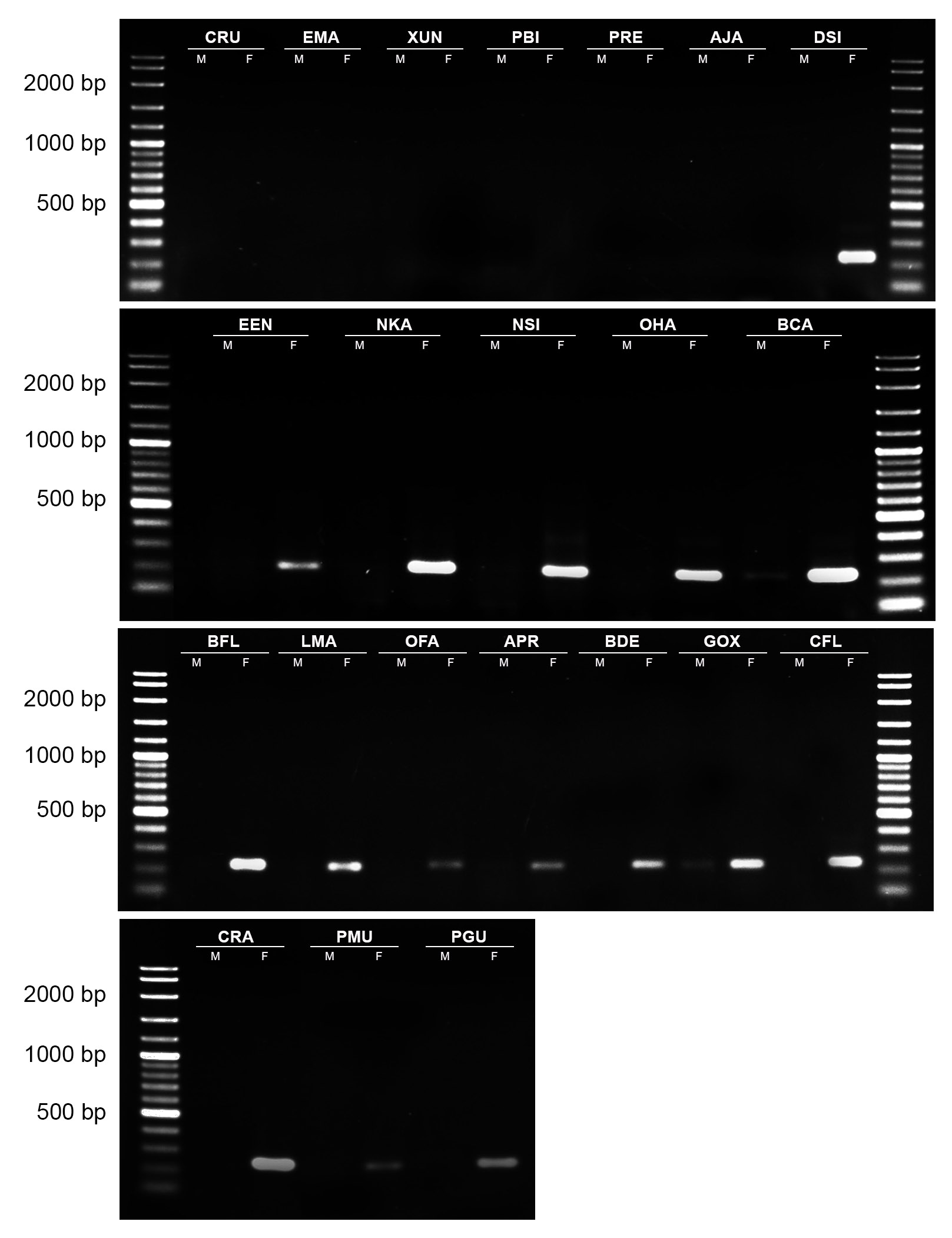

Supplement: Supplementary file 4 [file ECE3-7-4661-s004.jpg]

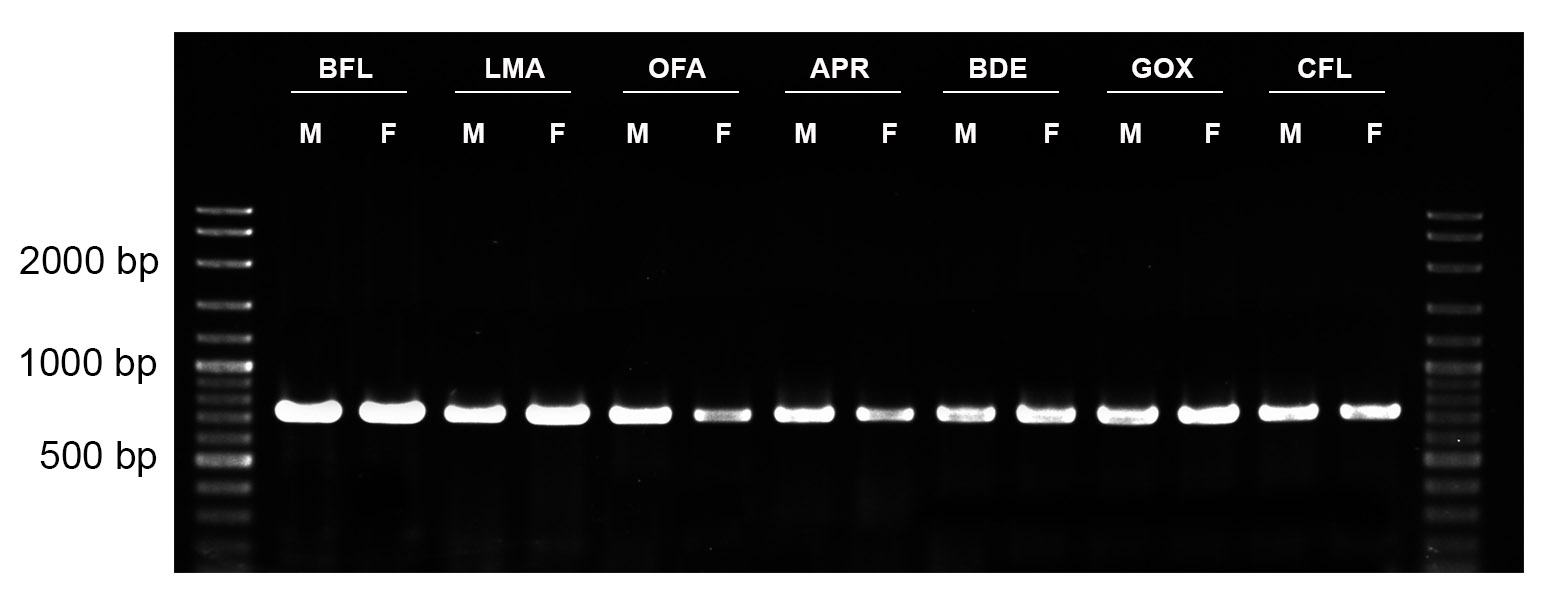

Supplement: Supplementary file 5 [file ECE3-7-4661-s005.jpg]
